# K_v_7 and K_2P_ Potassium Channels in Thalamocortical Function and Their Therapeutic Potential in Childhood Absence Epilepsy

**DOI:** 10.1002/ardp.70276

**Published:** 2026-06-09

**Authors:** Anushka Takhi, Guiscard Seebohm, Thomas Budde, Annika Lüttjohann

**Affiliations:** ^1^ Institute of Physiology I Münster University Münster Germany; ^2^ Myocellular Electrophysiology and Molecular Biology Münster University Münster Germany

## Abstract

Potassium (K^+^) channels are one of the key regulators of neuronal excitability and network stability. Among all the potassium channels, the voltage‐gated K_v_7 (*KCNQ*) and the two‐pore domain potassium (K_2P_) channels play an important role in stabilizing and maintaining the resting potential of neurons. They are involved in controlling the input resistance and in the generation of rhythmic activity in the thalamocortical network. The main functions of the thalamocortical system are the regulation of sensory processing and the generation of brain rhythms underlying sleep and wakefulness. By this, it is essential to many aspects of cognition. The thalamocortical system is explicitly sensitive to fine changes in potassium conductance. The dysregulation of thalamocortical oscillation, also known as thalamocortical dysrhythmia, has been known to be involved in several psychiatric and neurological disorders. A prototypical thalamocortical dysrhythmia is childhood absence epilepsy (CAE), in which hypersynchronous activity in the form of spike and wave discharges (SWD) induces sudden losses of conscious awareness in patients. This review will summarize the current knowledge on K_v_7 and K_2P_ channels in the regulation of synchrony and excitability in the brain and the thalamocortical system in particular, outlining future research directions, highlighting the therapeutic potential of these channels for the treatment of thalamocortical dysrhythmias in general and CAE specifically.

## Graphical abstract

Modulation of K_2P_ and K_v_7 channels has the potential to reduce hyperexcitability and hypersynchronicity within the thalamocortical system. This offers a new avenue to suppress spike and wave discharges, the electrophysiological hallmark of absence epilepsy.

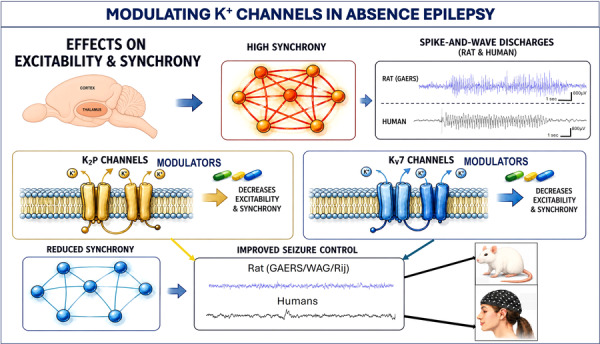

## 1 Introduction

The potassium (K^+^) channel superfamily is among the largest and most molecularly diverse family of ion channels, with > 70 human genes encoding a wide variety of channel proteins that are structurally and functionally distinct [1]. This large group of genes is diverse and gives rise to several subfamilies, including voltage‐gated (K_v_), inwardly rectifying (K_ir_), calcium‐activated (K_Ca_), and two‐pore domain (K_2P_) channels, each of which makes specific contributions to cellular electrophysiology [2]. These channels are characterized by their selective mediation of K^+^ ion efflux down their electrochemical gradient [3, 4]. This role is central to the establishment of the resting membrane potential, returning the membrane potential following depolarization, and the ongoing regulation of membrane voltage in a variety of excitable and non‐excitable cells [5]. Through this outward potassium conductance, K^+^ channels serve as a key determinant of cellular electrical stability while enabling dynamic responses to physiological stimuli [6, 7].

Within the nervous system, potassium currents play a key role in stabilizing the membrane potential and shaping the action potential dynamics, making them central regulators of neuronal excitability [8]. Voltage‐dependent gating and ion flux contribute to repolarization and firing adaptation, while broader channel expression across cell types highlights their functional ubiquity [9] Importantly, the wide diversity and differential expression of potassium channel subtypes among neuronal populations give rise to cell type‐specific electrophysiological properties of neurons. This is especially the case in the thalamus, where specific profiles of ion channel expression determine the intrinsic firing modes and functional specialization of individual classes of neurons [10].

Among the potassium channel families, the voltage‐gated K_v_7 channels (encoded by *KCNQ* genes) represent a key regulator for governing subthreshold excitability through the generation of the M‐current [11, 12]. This current is activated near the typical neuronal resting potentials of −60 to −70 mV, thereby modulating neuronal responsiveness. It positions K_v_7 channels as key determinants of neural circuit behavior and promising pharmacological targets for neurological disorders [12]. Structurally and mechanistically, *KCNQ* channels are phosphatidylinositol‐4,5‐biphosphate‐modulated voltage‐gated channels whose activity influences neuronal and cardiac physiology and whose mutation spectrum is strongly associated with neurological disease, including epilepsy [13].

Functionally, the M‐current mediated by K_v_7 channels increases as the membrane potential approaches firing threshold, contributes to the medium after‐hyperpolarization that limits burst activity, and opposes depolarizing currents promoting repetitive firing [12]. Due to the intrinsic burst‐generating properties of thalamocortical relay neurons, activation of K_v_7 and leak K_2P_ channels promotes the membrane hyperpolarization required for low‐threshold Ca^2+^ spikes and burst firing, while concurrently suppressing tonic firing at depolarizing potentials [14, 15, 16]. Modulation of K_v_7 conductance directly impacts neuronal output. Pharmacological boosting of native M‐currents curbs repetitive firing in sensory neurons, underscoring their function in stabilizing excitability throughout neural circuits [17].

In contrast to voltage‐gated potassium channels, two‐pore domain potassium (K_2P_) channels generate background “leak” currents that help to maintain the resting membrane potential without relying on traditional voltage sensors, and provide a steady current that holds the membrane at a negative voltage and resists depolarizing influences [18, 19]. K_2P_ channels are widely expressed in neuronal tissues. They stabilize the membrane potential through their hyperpolarizing leak conductance, which also modulates rhythmic neuronal firing [19]. This highlights their active role in network dynamics, far beyond just a passive background.

Together, voltage‐gated K_v_7 channels and voltage‐independent K_2P_ channels provide complementary control over neuronal excitability by combining K_v_7's dynamic voltage‐dependent gating along with K_2P_'s steady baseline conductance. These combined actions maintain resting membrane potential and regulate action potential generation by setting firing thresholds to prevent hyperexcitability. When these systems falter, it disrupts the electrical balance and can trigger aberrant network activity seen in neurological disorders like epilepsy [12, 19].

Understanding how K_v_7 and K_2P_ channels interact is key to figuring out how subtle molecular perturbations translate into circuit dysfunction, especially in brain networks tied to the generation of rhythmic physiological activity and its pathophysiological counterpart of hypersynchronous and hyperexcitable rhythms as seen in absence epilepsy. This review gathers the insights on their biology, spotlighting their roles in tuning excitability and contributing to dysfunctional network patterns seen in absence epilepsy.

## 2 The Thalamocortical Network and Its Role in Spike‐Wave Discharges in Childhood Absence Epilepsy (CAE)

**Figure 1:**
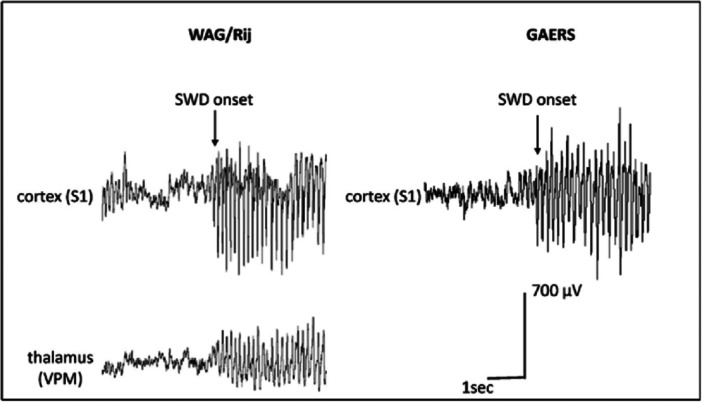
Representative local field potential (LFP) traces from the deep layer of S1 in a GAERS rat (right) and from the deep S1 and VPM of a WAG/Rij rat (upper and lower left, respectively). The arrows denote the onset of the spike‐wave discharges (SWD), identified following the criteria described by Van Luijtelaar and Coenen [24], where the onset corresponds to the peak of the first spike exceeding twice the baseline amplitude.

Absence epilepsy is a chronic neurological disorder affecting approximately 50 million people worldwide. It is characterized by short, non‐convulsive but frequent seizure episodes involving an abrupt loss of consciousness and brief episodes of behavioral arrest, which go along with a prototypical pattern of brain activity in the form of bilaterally synchronous and highly rhythmic spike and wave discharges (SWDs), which can be observed in an EEG of patients [20] (Figure 1). Work on two genetic rat models of absence epilepsy (GAERS and WAG/Rij rats), which have been proven to possess a high predictive validity to the human condition [21], showed that SWD are generated and maintained in the thalamocortical system of the brain. Moreover, a focal, hyperexcitable seizure onset zone was identified in the deep layers of the somatosensory cortex [22, 23].

Cortical pyramidal cells in S1 excite both thalamic relay neurons and GABAergic inhibitory neurons of the reticular thalamic nucleus (nRT/RTN). The nRT then, in turn, provides inhibitory feedback to thalamic relay neurons projecting to S1. By hyperpolarizing the membrane potential of the thalamic relay cells, they switch from a tonic into a burst firing pattern, which is seen to occur phase locked to the spike of the SWD [25, 26, 27].

It has been shown that the burst firing of thalamic relay neurons is carried by low‐threshold T‐type calcium channels (Cav3.x), and modulated by GABA_B_ receptor‐mediated inhibition and potassium conductance (K_v_7, K_2P_) [28, 29]. Previous studies using GAERS and WAG/Rij rats showed that altered expression or activity of potassium channels contributes to neuronal hyperexcitability in thalamic and cortical circuits by demonstrating that pharmacological blockade or genetic disruption of specific potassium channel subtypes enhances intrinsic firing and burst propensity of these neurons, resulting in more frequent and pronounced SWDs [30, 31].

To learn more about the mechanisms of CAE, genetic mutations and susceptibility loci are being investigated using molecular and animal models. Mutations in *CACNA1H, GABRG2*, and *KCNQ2/3* genes facilitate rhythmic bursting activity [32]. Together, these findings illustrate that a finely tuned interplay between cortical excitation, thalamic bursting, and RTN‐mediated inhibition governs the initiation and termination of SWDs in absence epilepsy.

## 3 Two Pore Domain (K_2P_) Channels: Molecular Features and Brain Distribution

**Figure 2:**
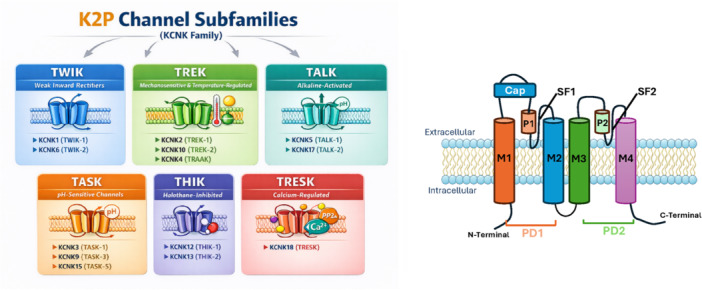
K_2P_ channel family relationships and structure. (Left) K_2P_ family classification. Subfamilies and key characteristics are indicated. (Right) K_2P_ subunit diagram. Pore domains 1 and 2 (PD1 and PD2), transmembrane helices (M1–M4), pore helices (P1 and P2), selectivity filters (SF1 and SF2), and the Cap domain are indicated.

The two‐pore‐domain potassium (K_2P_) channels are the background “leak” potassium channels that maintain the resting membrane potential and excitability of neurons [33]. These K_2P_ channels comprise subunits that each consist of four transmembrane segments and two pore‐loop (P) domains with intracellular amino‐ and carboxyl‐termini (4TM/2P) (Figure 2). Two such subunits dimerize so that the four P loops form a single K^+^ selective conduction pathway [34]. They function as homo‐ or heterodimers rather than tetramers, as often seen in other potassium channels [35]. They are also called “leak” potassium channels, as they are active over a wide voltage range and contribute to voltage and time‐dependent background currents [36]. The K_2P_ subunits form a dimeric assembly. In the central nervous system, K_2P_ channels are expressed in multiple brain areas, including the dorsal root ganglion (DRG), the trigeminal ganglion (TG), the spinal cord, cerebrum, cerebellum, and the thalamus. In the peripheral nervous system, they are mainly expressed in sensory neurons from the DRG and TG [37]. In the central nervous system, K_2P_ channels are not confined to a single cell type but are expressed in neurons and glia, with overlapping yet distinct subtype profiles. Neuronal membranes mainly express TREK‐2 (K_2P_10.1), TRAAK (K_2P_4.1), TWIK‐1 (K_2P_1.1), TRESK (K_2P_18.1), TASK‐1 (K_2P_3.1), TASK‐3 (K_2P_9.1), and TREK‐1 (K_2P_2.1), where they provide background K^+^ conductance and help set excitability, firing patterns, and synaptic integration [19, 38]. In contrast, glial K_2P_ expression is dominated by TWIK‐1 (K_2P_1.1) and TREK‐1 (K_2P_2.1) in astrocytes, alongside additional K_2P_ subtypes in oligodendrocytes and microglia that shape glial membrane potential, K^+^ buffering, and neuroinflammatory signaling [19, 39, 40].

The mammalian K_2P_ family comprises 15 *KCNK* genes, which are divided into at least six functional subgroups—TWIK, TREK, TASK, TRESK, TALK, and THIK (Figure 2). Each subgroup has different gating stimuli, such as pH, lipids, temperature, mechanical strength, and tissue distribution [41]. The K_2P_ channels possess a distinctive extracellular cap that is formed by outer helices and loops that create side lateral pathways, thought to shape ion access and pharmacology while covering the central pore entrance [41]. Due to their background conductance and multiple regulatory inputs, K_2P_ channels shape neuronal firing patterns, sensory transduction, vascular tone, and hormone secretion [42, 43]. These channels have also been implicated in disorders including pain, depression, sleep disorders, epilepsy, and migraine, making these channels an attractive therapeutic target [36].

### 3.1 K_2P_ Channels in Thalamocortical Circuits Relevant to Absence Seizures

The K_2P_ channels provide the major background K^+^ conductance in cortical pyramidal neurons, thalamic relay cells, and inhibitory interneurons, thereby maintaining the resting membrane potential and input resistance in the thalamocortical loops [19]. Luo et al. summarize expression of the K_2P_ subfamilies (e.g., TASK, TREK, TWIK) in thalamus and cortex, mentioning that both excitatory relay neurons and GABAergic neurons (including reticular and cortical interneurons) express K_2P_ channels that contribute to their resting conductance and responsiveness to synaptic inputs [19, 44]. In the thalamus, the activity and modulation of TASK and TREK channels are key to the switch from burst to tonic firing in thalamic neurons and shaping rhythmic sleep‐related spindling [14, 45].

Currently, unlike K_v_ and K_Na_ genes, no K_2P_ genes have been established as a monogenic cause of the typical CAE. Rather, K_2P_ channels are viewed as modulators of generalized seizure susceptibility due to their impact on resting conductance and network excitability [19]. Absence seizures represent hypersynchronous cortico‐thalamo‐cortical oscillations [19, 46]. Channels that maintain the membrane potential and shape rebound bursting, such as K_2P_ leak channels, can influence the tendency for spike‐wave discharges, even though K_2P_‐specific absence epilepsy models remain sparse. A review by Bista et al. [14], highlights that K_2P_ channels (TASK/TREK) regulate background K^+^ currents in the thalamocortical relay neurons, contributing substantially to the standing outward K^+^ current as evidenced by voltage‐clamp ramps (pH sensitivity: ~35%–40% inhibition at pH 6.0), pharmacology (e.g., A293 for TASK, spadin for TREK), and Gq‐coupled muscarinic modulation. These channels stabilize resting potential and burst‐to‐tonic firing switches, positioning K_2P_ channels to modulate 2.5–4 Hz spike‐wave discharge timing, stability, and propagation, despite limited direct causal evidence [14]. A broad number of CNS disease studies, particularly Bista et al. [14] on thalamic K_2P_ channels in epilepsy models and reviews like Luo et al. (2021), propose K_2P_ channels (e.g., TASK‐1//3, TREK‐1) as therapeutic targets. Pharmacological openers enhancing the background leak currents could possibly dampen the thalamocortical synchrony seen during absence seizures, for example, via hyperpolarization stabilizing burst firing, whereas the inhibitors might worsen the generalized seizure risk by promoting depolarization and hyperexcitability [14, 19]. However, targeted trials on K_2P_ channel modulation studies for absence seizure generation are not yet available, leaving their role primarily as mechanistic modifiers rather than validated drug targets [44].

Beyond the contribution of K_2P_ channels alone, the interplay between K_2P_‐dependent leak currents and HCN channel‐dependent inward current is also of particular importance for the regulation of thalamocortical excitability and oscillatory activity [47, 48]. K_2P_ channels produce a background potassium conductance that helps to stabilize the resting membrane potential and modulate the input resistance, and HCN channels generate *I*_h_, a depolarizing inward current activated by membrane hyperpolarization [38, 49]. Thus, the relative balance of these two conductances determines the voltage range of the membrane in which thalamocortical neurons switch from tonic to burst firing modes [47, 50]. In the context of absence epilepsy, altered K_2P_ activity could modify HCN channel activation by changing baseline membrane potential and membrane responsiveness, thereby influencing rhythmic oscillations in thalamocortical circuits and spike‐wave discharge generation [48, 51, 52]. Thus, K_2P_ and HCN channels should be considered as functionally interacting regulators of thalamocortical network dynamics rather than as independent contributors to neuronal excitability [49, 52].

### 3.2 Modulation of K_2P_ Channels as an Emerging Strategy

The two‐pore domain potassium channels function as “leak” channels, which maintain the stable resting membrane potential of neurons and glia while they regulate the entire nervous system's cellular excitability [19, 34]. A study conducted by Luo and his team in 2021 examined how K_2P_ channels function under normal conditions and how their malfunctioning leads to neurological disorders, which include CAE, along with chronic pain, ischemia, sleep problems, and depression. Neuronal K_2P_ channels in healthy brains, including TREK‐1 and TASK‐1/3, generate background hyperpolarizing currents that reduce excessive neural firing, while glial K_2P_ in astrocytes enable potassium buffering and glutamate homeostasis to protect against excitotoxicity [19]. Loss of K_2P_ channel function in sensory neurons reduces K^+^ efflux, increasing neuronal excitability, heightening pain sensitivity, and contributing to network hyperexcitability in epilepsy models [19]. The thalamocortical pathway in CAE shows that K_2P_ channel modifications, particularly TASK channels, serve as a fundamental mechanism behind the rhythmic spike‐wave discharges that define absence seizures, thus making these channels important for future research [19].

**Figure 3:**
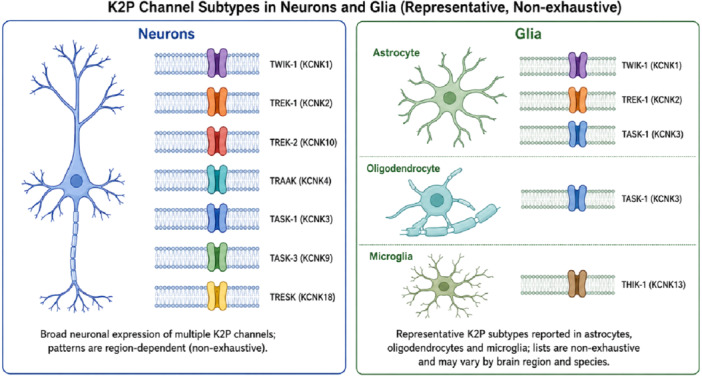
Representative distribution of two‐pore‐domain potassium (K_2P_) channel subtypes in neurons and glia. The left panel illustrates a cortical neuron expressing multiple K_2P_ channels, including TWIK‐1 (*KCNK1*), TREK‐1 (*KCNK2*), TREK‐2 (*KCNK10*), TRAAK (*KCNK4*), TASK‐1 (*KCNK3*), TASK‐3 (*KCNK9*), and TRESK (*KCNK18*), reflecting the broad and region‐dependent neuronal expression of this channel family. The right panel depicts major glial cell types and their reported K_2P_ subtypes: astrocytes (TWIK‐1, TREK‐1, TASK‐1), oligodendrocytes (TASK‐1), and microglia (THIK‐1*/KCNK13*). Together, these schematics highlight that both neuronal and glial K_2P_ channels contribute to background potassium conductances across diverse brain cell populations [19].

In Figure 3, the distribution of K_2P_ channel subtypes in neurons and glial cells is shown in separate compartments. Although direct data from absence epilepsy models are still limited, it seems likely that modulating K_2P_ channels will have different consequences in neurons and glial cells: neuronal K_2P_ activity mainly affects thalamocortical excitability and burst‐tonic transitions, whereas glial K_2P_ activity influences extracellular K^+^ homeostasis and gliotransmission, which can in turn shape the emergence and stability of spike‐wave discharges [19]. Importantly, the contribution of glia cells to ictogenesis and epileptogenesis of absence epilepsy modulation has recently been highlighted, while additional studies to elucidate neuron–glia interactions are still ongoing [53].

Research by Busserolles et al. [54] and M. Chen et al. [55] demonstrated that TREK‐1/TASK activators showed effective results in preclinical studies for pain management through their ability to reduce hyperexcitability, which may also suggest a potential for seizure control [54, 55]. Recently, the activation of K_2P_ channels as an effective anti‐epileptic mechanism has been linked to TRESK channels [56]. Seizure‐induced short‐term plasticity of hippocampal neurons, leading to the up‐regulation or the experimental genetic overexpression of TRESK channels, attenuates temporal lobe epilepsy. However, to date, no study has attempted to directly target K_2P_ channels for the treatment of absence epilepsy, despite the obvious therapeutic potential of these channels as pharmacological openers [57]. This gap represents a pivotal opportunity, as K_2P_ channel sensitivity to pH, temperature, stretch, lipids, and GPCR signaling provides multiple avenues for selective activation [58].

K_2P_ widespread expression in various body tissues poses a significant obstacle, highlighting the need for drugs that target specific subtypes to improve treatment outcomes and minimize side effects. This approach has the potential to revolutionize treatments for CAE and other disorders. The study by Luo et al. (2021) shows that K_2P_ functions as an important new treatment target that researchers should investigate further to develop targeted therapies, particularly for absence epilepsy, which requires treatment options that currently do not exist [19].

## 4 Voltage‐Gated Potassium (K_v_7‐ *KCNQ)* Channels and the M‐Current

**Figure 4:**
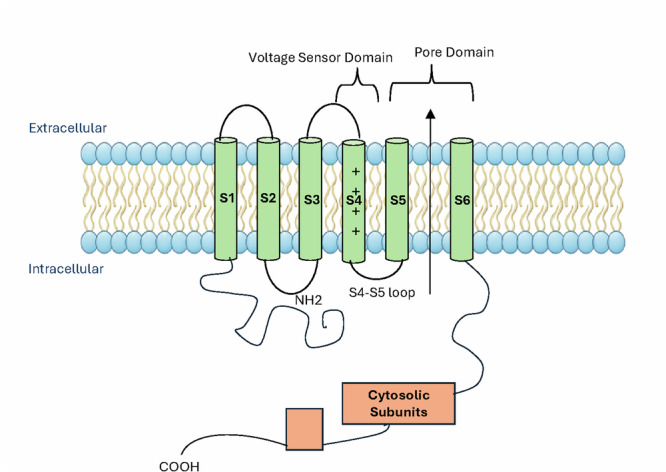
Schematic representation of one K_v_7 channel subunit. These channels have six transmembrane domains (S1–S6) per subunit, with the S4 segment acting as a voltage‐sensing domain and S5–S6 forming the pore domain. The intracellular N‐ and C‐terminal mediate subunit interactions and PIP_2_ binding, which is essential for channel activation and stability.

Voltage‐gated potassium channels are key players in both restoring the resting membrane potential after an action potential and in limiting action‐potential generation in neurons, thereby shaping the electrical responses of excitable cells such as neurons and heart muscle cells. The human genome contains 40 voltage‐gated potassium channels, organized into 12 subfamilies [59, 60]. Among these families, the *KCNQ* family consists of five members, *KCNQ1‐5*, which are also named as K_v_7.1–K_v_7.5 [60]. These voltage‐gated channels influence the action potential initiation and propagation, especially the K_v_7 subunits K_v_7.2, K_v_7.3, and K_v_7.5, which are widely expressed across excitable cells. The *KCNQ2, KCNQ3*, and *KCNQ5* genes are known to encode for the subunits of the K_v_7.2, K_v_7.3, and K_v_7.5 potassium channels, respectively [61, 62]. The neuronal K_v_7 channels are widely expressed in the peripheral and central nervous system. These assemble as homotetramers or heterotetramers (e.g., K_v_7.2/K_v_7.3 for M‐current), with each α‐subunit containing six transmembrane domains, with the S4 segment acting as a voltage sensor and S5–S6 forming the pore [63] (Figure 4). Recent structural studies, including cryo‑EM analyses of voltage‑gated potassium channels such as K_v_7.1, indicate that subtle shifts in the S4–S5 linker can modify the coupling between the voltage‑sensing domain and the pore, thereby altering channel activation and deactivation kinetics [64]. The cryo‑EM structure of *KCNQ4* (K_v_7.4) provides important mechanistic and pharmacological insights into the K_v_7 family, revealing conserved structural features among the neuronal K_v_7.2–7.5 subunits while highlighting key differences compared to K_v_7.1. These structural and pharmacological distinctions, together with the observation that K_v_7.2–7.5‑selective openers such as retigabine do not activate K_v_7.1, support the feasibility of developing subtype‑selective K_v_7 modulators tailored to neuronal (K_v_7.2–7.5‑rich) versus non‑neuronal (K_v_7.1‑rich) channel formations [65, 66]. These structural templates offer a rational basis for designing subunit‑specific drugs with improved selectivity, potentially transforming therapeutic approaches for epilepsy, cardiac arrhythmias, and neuropathic disorders [66].

K_v_7 channels generate an outward current, termed M‐current (*I*_M_), that exerts strong control over neuronal excitability and reveals characteristics that make it distinct from other voltage‐gated potassium channels [1]. The M‐current is non‐inactivating and inhibited following the stimulation of muscarinic acetylcholine receptors (M1 and M3), which features its name [67, 68]. These channels are activated near the resting membrane potential, therefore providing an outward current stabilizing the membrane potential and hence limiting hyperexcitation [67, 68]. Activation of Gq‐coupled receptors, such as muscarinic acetylcholine receptors (M1 and M3), suppresses the M‐current, resulting in membrane depolarization and enhanced neuronal excitability. By modulating the resting membrane potential, spike threshold, firing frequency, and afterhyperpolarization, K_v_7 channels play an important role in how neurons generate and regulate their electrical activity [61, 69, 70]. Neuronal K_v_7 channels are strategically positioned at the sites critical for action potential initiation, particularly at the distal axon initial segment (AIS), where they exert strong control over intrinsic excitability [12]. Immunohistochemical and live imaging studies show that K_v_7.2/7.3 are highly enriched at the AIS and nodes of Ranvier, colocalizing with Nav channels via a shared ankyrin‐G‐binding motif that anchors them to the AIS scaffold [71, 72]. This localization allows AIS K_v_7 channels to generate a subthreshold, non‐deactivating M‐current that hyperpolarizes the axonal membrane, raises action potential threshold, and limits spontaneous and repetitive firing. Conversely, blocking of the M‐current at the AIS lowers spike threshold and increases inherent firing, highlighting AIS‐enriched K_v_7 channels as a key brake on action potential initiation [12, 71]. The K_v_7 channels are pharmacologically modulated by blockers (e.g., XE991) and openers (e.g., Retigabine), with the latter forming the basis for a clinical use as an antiepileptic therapy [73]. Reduced K_v_7 channel activity or protein expression increases susceptibility to absence seizures, whereas channel activation by pharmacological agents can decrease seizure propensity and may provide a promising therapeutic approach for absence epilepsy [74].

### 4.1 K_v_7 Channel in Thalamocortical Circuits Relevant to Absence Seizures

Studies of thalamic relay neurons and cortical pyramidal cells have demonstrated the role that K_v_7/M‐type potassium channels (*KCNQ2/3*) play in regulating the excitability of thalamocortical neurons by providing a non‐inactivating, slowly activating outward current that reduces repetitive firing [75]. The study carried out by Singh et al. [76] demonstrated that orthologous knock‐in mouse models carrying precise human mutations in the *KCNQ2* (Kv7.2) and *KCNQ3* (Kv7.3) subunits exhibit spontaneous generalised tonic‐clonic seizures and frequent generalised inter‐ictal cortical discharges, confirming the essential role of these M‐current channels in controlling neuronal excitability. Reduced K_v_7 channel activity increases cortical excitability, thereby promoting thalamocortical network oscillations that manifest as SWDs characteristic of absence seizures [12, 77, 78].

Thalamocortical rhythmicity and seizure susceptibility are altered in genetic models with *KCNQ2/3* mutations, suggesting that K_v_7 channel function contributes to the equilibrium between ictal and interictal activity in circuits linked to absence seizures [79, 80]. In experimental models, pharmacologically manipulating K_v_7 channels with blockers (like XE991) or activators (like retigabine/ezogabine) can change cortical synchronous activity linked to absence‐like paroxysms and modulate thalamocortical network excitability [74]. A study carried out by Oh et al. [81] showed that loss of Ank2 (ankyrin‐B) in cortical and hippocampal excitatory neurons produces a K_v_7.2/7.3 channelopathy that markedly increases cortical excitability and causes generalized seizures and juvenile seizure‐related death. K_v_7 channels, highly enriched at the AIS of corticothalamic and corticocortical neurons, normally provide a brake on repetitive firing and help to stabilize thalamocortical rhythms implicated in SWDs of absence epilepsy. In Ank2‐cKO mice, AIS lengthening and reduced K_v_7 density weaken this brake, while the K_v_7 agonist retigabine restores M‐current‐dependent afterhyperpolarization and normalizes cortical excitability, supporting K_v_7 enhancement along corticothalamic pathways as a rational strategy to dampen absence‐related thalamocortical oscillations [81]. Although K_v_7 channel modulators have anticonvulsant potential in generalized epilepsies, their effectiveness on absence seizures is not yet fully investigated. Since the K_v_7 channel expression and function change during development, which may influence the age‐dependent onset of absence epilepsy [82]. Their effectiveness may also rely on the particular thalamocortical state and genetic background [74, 82].

## 5 K_2P_ and K_v_7 Channels as Promising Therapeutic Targets

K_2P_ channels (TASK‐1/3, TREK‐1/2) provide leak K^+^ conductances that shape the thalamocortical neuron's resting potential, input resistance, and the switch between tonic firing (faithful relay information during wakefulness) and burst firing (associated with sleep or pathological states like absence seizures) [47]. Most thalamic data on epilepsy come from sensory relay nuclei in absence seizure models. A number of selective TASK blockers (such as A1899 and A293) and the respiratory stimulant doxapram inhibit TASK currents. TREK and TRAAK channels are sensitive to commonly used drugs, including volatile anesthetics, local anesthetics like bupivacaine, and antidepressants such as fluoxetine [41, 83]. More recently, small‐molecule TREK activators (e.g., ML67‐33) have shown that enhancing K_2P_‐mediated leak currents can stabilize membrane potential and dampen neuronal excitability [84, 85]. Together, these compounds illustrate how K_2P_ channels can be pharmacologically targeted to shift thalamocortical excitability and potentially modify spike‐wave discharges, complementing the insights gained from TASK‐3 mutations [19, 41, 43, 83]. To the best of our knowledge, to date, no K_2P_‐channel‐targeting drug has advanced to clinical use for absence epilepsy, and current evidence in this indication remains confined to preclinical models [57].

In contrast, K_v_7 activators like retigabine show broad anti‐seizure effects across focal (e.g., neocortical/hippocampal) and generalized epilepsies, while K_2P_ modulation may be more circuit‐specific [47, 86, 87]. However, retigabine's clinical development also showed important limitations; following approval for the treatment of partial‐onset focal seizures, it was withdrawn again in 2017 due to safety concerns, including pigmentation changes and urinary adverse effects [88, 89]. These observations raise a reasonable concern that follow‐on K_v_7 channel activators may share a class effect, especially if they retain the same basic chemical scaffold or produce similar tissue profiles. For this reason, any new K_v_7 activators will need to preserve efficacy while demonstrating improved selectivity, pharmacokinetics, and tolerability to avoid repeating the liabilities observed with retigabine [90]. Several next‐generation K_v_7 activators, including BHV‐7000 (opakalim), QRL‐101, azetukalner (XEN1101), and retigabine‐derived analogs such as SF0034 are now in development to retain antiseizure efficacy while improving selectivity, stability, and safety, addressing the concerns of retigabine's class‐effect toxicities through better molecular design that reduces off‐target exposure [74, 90, 91]. K_v_7 activators are approaching the clinic as potential antiseizure agents, their precise efficacy and safety profile in typical absence epilepsy will need to be clarified in dedicated future studies [74, 92].

Retigabine stabilizes K_v_7 channels in the open state and shifts their activation curve toward more hyperpolarized potentials, thereby increasing M‐current amplitude at rest [93]. Consistent with this mechanism, retigabine reduces neuronal firing probability and exhibits antiseizure effects in several preclinical models, including status epilepticus and focal and generalized seizures [15, 16, 86]. In WAG/Rij rats, a genetic model of typical absence epilepsy, retigabine modifies SWD parameters in an age‐ and dose‐dependent manner, indicating that K_v_7 modulation can directly influence absence‐like spike‐wave activity [94]. In this study [94], WAG/Rij rats were given systemic (intraperitoneal) retigabine, which increased the number and duration of SWDs in aged animals, indicating a potential pro‐epileptic effect in this model. A similar aggravation of non‐convulsive seizures has been reported for the experimental K_v_7 activator IDOR‐1104‐0086 [95] in an absence‐like rat model. These findings emphasize that dose and age will be critical in determining whether K_v_7 activation is beneficial or detrimental in typical absence epilepsy. Moreover, network state and thalamocortical connectivity might form crucial determinants for the effectiveness of these modulators underlining the need for careful, model‐specific evaluation of next‐generation K_v_7 activators in this seizure type [94, 95]. For example, it remains possible that more targeted delivery strategies, like local administration of K_v_7 activators to specific cortical or thalamic nodes within the spike and wave generating network, might stabilize these circuits and reduce absence seizures. This hypothesis will require systematic testing in future studies comparing focal versus systemic dosing paradigms [96, 97].

Overall, these observations suggest that K_v_7 channel activation can either facilitate thalamic bursting by hyperpolarizing neurons sufficiently to de‐inactivate T‐type Ca2+ channels or suppress network activity via strong shunt and reduced responsiveness, with net antiseizure effects likely emerging when the system is pushed outside its oscillation‐permissive regime [15, 16, 87].

Absence seizures rely on finely tuned intrinsic currents (including K_2P_ leak) and synaptic interactions that drive hypersynchronous SWDs [87, 98]. Computer modeling and experimental work indicate that robust rhythmic bursting in thalamocortical neurons requires an optimal combination of at least seven different subthreshold conductances, including leak K_2P_ conductance, low‐threshold Ca^2+^ current, and several inward and outward voltage‐ and Ca^2+^‐dependent currents [47, 99, 100, 101]. These include a persistent Na^+^ current that supports slow depolarization between bursts and hyperpolarization‐activated cyclic nucleotide‐gated (HCN) channels, whose inward current helps pace rebound depolarization after hyperpolarizing phases [102]. Ca^2+^‐activated K^+^ currents are also important players: small conductance (SK) channels generate slow, long‐lasting after‐burst hyperpolarization that limits burst duration and burst probability, thus providing a brake on thalamic and cortical hyperexcitability [103, 104, 105]. Large conductance (BK) channels mediate fast, high conductance repolarization associated with presynaptic Ca^2+^ entry and repolarization of the action potential, thereby shaping spike timing. In this respect, it is interesting to note that BK channels differentially influence bursting in epileptic and non‐epileptic rats [106]. Na^+^ and other K^+^ conductances further refine the depolarization and repolarization cycles during oscillatory activity [107].

With the broad availability of state‐ and subtype‐specific potassium channel modulators and based on the fact that K^+^ conductances are generally stabilizing, gain dampening components of cellular and network activity, modulation of potassium channels particularly subthreshold K_v_7 and background K_2P_ conductances is likely to provide a more targeted and potentially safer therapeutic strategy than directly targeting inward Ca^2+^ or Na^+^ channels, which carry a higher risk of disrupting excitation‐inhibition balance or provoking pro‐arrhythmic or pro‐ epileptic states [74, 108].

In this framework, moderate inhibition of TASK/TREK channels depolarizes thalamocortical neurons, shifts them out of the voltage range that favors low‐threshold bursting, and can therefore diminish SWD‐related rhythmicity even though overall excitability may rise [14, 38]. Conversely, strong activation of K_2P_ channels can hyperpolarize and shunt thalamocortical neurons so effectively that action potential generation is suppressed, which also disrupts the thalamocortical synchrony needed to sustain SWDs [87]. These bidirectional effects support the concept that both decreasing and increasing K_2P_ activity, depending on baseline channel function and network state, may be anti‐oscillatory and thus potentially anti‐absence [47, 87].

Most mechanistic work on TASK and TREK in the thalamus has focused on thalamocortical relay neurons, where muscarinic and other G‐protein‐coupled receptors dynamically inhibit K_2P_ conductances via PIP_2_ depletion and DAG generation to promote depolarization and firing‐mode switches [14, 37, 47]. However, K_2P_ channels are also expressed in other thalamic cell types, including neurons of the nRT and various interneuron populations [109], where they are expected to modulate inhibitory output and thus shape thalamocortical synchrony in a cell‐type‐specific manner [47]. The precise contribution of K_2P_ channels in nRT neurons and local interneurons to SWD generation and termination remains largely unresolved and represents a key question for future work [87].

Pharmacological and genetic studies in rodent models support a broader role of TREK and TASK channels in seizure susceptibility and network excitability, although direct testing of selective K_2P_ activators or inhibitors in validated absence epilepsy models is still scarce [47]. Some K_2P_ channels, particularly TASK‐3 (*KCNK9*), have been directly linked to absence epilepsy in both people and animal models [110, 111]. In GAERS, Holter and colleagues described a TASK‐3 mutation, an extra alanine in a C‐terminal polyalanine stretch at a locus corresponding to human 8q24, which has been associated with CAE [110]. Even though this variant does not cause a dramatic change in leak K^+^ current in heterologous systems or thalamic neurons, it supports the idea that relatively subtle alterations in K_2P_ function or regulation can increase the vulnerability of corticothalamic networks to absence seizures [19, 110]. Clinically and experimentally, spike‐and‐wave discharges tend to appear during slightly reduced vigilance and are associated with a comparatively hyperpolarized membrane potential in thalamocortical neurons [57, 112]. In this hyperpolarized regime, K_2P_‐mediated leak currents are well placed to fine‐tune resting potential and the switch between tonic and burst firing, and thereby to influence whether spike‐and‐wave discharges emerge and how stable they are [19, 47]. Beyond genetic variation in TASK‐3, pharmacological modulation of K_2P_ channels offers a complementary way to probe their role in absence epilepsy [19, 83]. Selective TREK‐2 activators hyperpolarize DRG nociceptors and reduce ${\text{Ca}}^{2+}$ influx, illustrating how K_2P_ enhancement may also dampen high‐frequency firing in central neurons as well [113]. In the thalamus, modulation of leak potassium currents alters rhythmic burst firing patterns linked to SWD generation, implying that carefully titrated K_2P_ modulation could shift thalamocortical circuits away from pathological synchrony without abolishing physiological oscillations required for sleep and cognition [14, 47, 100, 101].

The Genetic Absence Epilepsy Rat from Strasbourg (GAERS) and the Wistar Albino Glaxo rats from Rijswijk (WAG/Rij) are well‐validated genetic models of typical absence epilepsy that display spontaneous SWDs closely resembling human absence seizures [114, 115]. Both strains show stable, pharmacologically responsive thalamocortical oscillations, but they differ in SWD frequency, duration, and associated behavioral and comorbid profiles, which need to be considered when testing ion‐channel‐targeted therapies [21]. Recent multi‐omic analyses in GAERS have revealed widespread alterations in metabolic and signaling pathways in the somatosensory cortex and thalamus, underlining that channel dysregulation occurs within a broader network and molecular context [116]. These models, therefore, offer suitable platforms to probe how baseline K_2P_ and K_v_7 channel expression and function interact with targeted modulators to influence absence seizure propensity [21, 116].

In absence epilepsy, reducing pathological thalamocortical excitability and synchrony via modulation of intrinsic potassium conductance is a mechanistically coherent approach [87]. For K_2P_ channels, both inhibition (to depolarize thalamocortical neurons and prevent optimal low‐threshold bursting) and strong activation (to hyperpolarize and shunt neurons) are plausible anti‐oscillatory strategies whose efficacy and safety likely depend on cell type, brain region, and disease stage [14, 47]. For K_v_7 channels, moderate activation may dampen excessive cortical and thalamic excitability and reduce SWD burden, whereas stronger activation could silence thalamic relay activity and disrupt physiological rhythms, emphasizing the need for finely tuned modulation [87, 94].

## 6 Conclusion and Outlook

The current data support that K_2P_ and K_v_7 channels are promising but complex therapeutic targets whose impact on absence seizures depends critically on the location and degree of modulation within thalamocortical circuits. Effective anti‐absence strategies will entail the adjustment of K_2P_ and K_v_7‐mediated conductance in such a way that the narrow parameter range of slight hyperpolarization of thalamocortical neurons favoring burst firing is avoided. As outlined above, spike‐and‐wave discharges tend to appear during slightly reduced vigilance and are associated with a comparatively hyperpolarized membrane potential in thalamocortical neurons [57, 112]. In this hyperpolarized regime, K_2P_‐mediated leak currents are well placed to fine‐tune resting potential and the switch between tonic and burst firing, and thereby to influence whether spike‐and‐wave discharges emerge and how stable they are [19, 47]. Data from the current review demonstrate that this might be achievable by either a moderate reduction of K_2P_‐mediated leak currents or by an enhancement of K_v_7 activity, which allows re‐establishing a resting potential balance that ensures a controlled depolarization of thalamocortical neurons and thus prevents their entry into the hyperpolarized voltage range that promotes burst firing, favoring SWD. As rhythmic bursting is thought to be associated with spike‐wave discharge generation, inhibition of the underlying mechanisms may dampen spike‐wave activity, even though direct pharmacological studies of K_2P_ activators in validated absence epilepsy models are still scarce. Under various baseline conditions, both increasing and decreasing K_2P_ activity, as well as carefully titrated K_v_7 activation, may be advantageous; strong activation may exert shunt‐like inhibition that eliminates firing.

Future studies can utilize GAERS and WAG/Rij rats as a translational model to investigate how the augmentation of K_v_7 M‐currents and K_2P_ leak currents affects thalamocortical network activity during pre‐ictal, ictal, and interictal periods. By integrating chronic multi‐site local field potential (LFP) recordings with behavioral observations, one can directly correlate the modulation of intrinsic potassium currents with the characteristics of SWD and subtle behavioral manifestations of absence seizures.

With the help of selective K_v_7 and K_2P_ modulators (such as TREK, TASK, and TRESK modulators), it is possible to examine whether the enhancement of these “brake” currents not only decreases the amount and duration of SWD episodes but also corrects the pathological coupling patterns and pre‐ictal network features that are associated with cognitive and affective comorbidities. The same set of analytical tools (phase‐amplitude coupling, non‐linear association measures, Granger causality) can be used to explore channel‐specific “network fingerprints” that could, in the long run, be used as biomarkers of target engagement in patients.

Given the well‐characterized channelopathies and reproducible thalamocortical oscillations in GAERS and other lines such as WAG/Rij, these animals are ideal for exploring cell‐type and circuit‐level effects of K_v_7 and K_2P_ modulation in slice patch‐clamp and in vivo electrophysiology. This research can, therefore, close the gap between biophysical cell physiology (modulation of M‐currents and leak currents), mesoscopic dynamics (thalamocortical synchrony and SWD onset/termination), and macroscopic outcomes (seizure frequency, motor symptoms, and network markers of comorbidity).

In the long run, pharmacology specific to the channels could be combined with the current treatment options for absence seizures in a rational polytherapy approach. The aim would be to decrease drug resistance and, at the same time, limit cognitive and mood‐related side effects. Instead of just controlling seizures, future treatment options may focus on stabilizing thalamocortical membrane potential dynamics more physiologically. Looking ahead, one of the most promising and as‐yet uncharted avenues is the potential for the early modulation of native potassium conductance to reorganize the developmental thalamocortical circuitry and halt the progression of absence epilepsy. Future studies might examine the impact of K_v_7 and K_2P_ modulation on learning behavior, attentional control, and stress response, areas that are frequently, but not fully understood, impacted in absence epilepsy.

Ultimately, through the integration of ion channel biophysics with circuit synchronization and behavior, this research could lead the way to mechanism‐based, circuit‐stabilizing therapies that not only control absence seizures but also enhance the quality of life and cognitive function.

## Conflicts of Interest

The authors declare no conflicts of interest.

## Data Availability

The data that support the findings of this study are available from the corresponding author upon reasonable request.
